# Nonverbal correlates of paranoid ideation – a systematic literature review

**DOI:** 10.1017/S0033291725001230

**Published:** 2025-06-04

**Authors:** Ron Haarms, Hannah E. Jongsma, Chris N. W. Geraets, Wim Veling

**Affiliations:** 1 University Centre for Psychiatry, University Medical Centre Groningen, Groningen, The Netherlands; 2 Centre for Transcultural Psychiatry Veldzicht, Balkbrug, The Netherlands

**Keywords:** behavioral measures, nonverbal correlates, paranoid ideation, psychosis, psychotic disorder

## Abstract

**Background and Aim:**

Insight in nonverbal correlates of paranoid ideation can potentially help improve diagnostic procedures and guide interventions. The aim was to systematically evaluate the scientific evidence investigating nonverbal correlates of paranoid ideation.

**Methods:**

The review follows the PRISMA guidelines. Databases PsycINFO, PubMed, Web of Science, and Cinahl were searched for studies concerning the use of standardized instruments for both verbal and nonverbal measurements of paranoid ideation in adult participants. Quality of studies was evaluated using the Effective Public Health Practice Project tool. Data were systematically extracted and summarized thematically and narratively. This review was registered with PROSPERO (CRD42022288001).

**Results:**

The search strategy yielded 3962 results of which 22 papers met inclusion criteria. Half (*n* = 11) of the included articles included patients with a diagnosis on the psychosis spectrum, the other articles (*n* = 11) studied healthy populations. Identified nonverbal categories were *spatial behavior* (*n* = 6)*, brain region activity* (*n* = 5)*, visual perception* (*n* = 5)*, stress physiology* (*n* = 4)*, information processing* (*n* = 3), and *aggression* (*n* = 1). Some studies investigated multiple nonverbal categories.

**Conclusions:**

Evidence was strongest for spatial behavior and brain region activity as nonverbal correlates of paranoid ideation. Evidence for stress physiology, information processing, and aggression as potential nonverbal correlates was less robust, due to inconsistent findings and small numbers of publications. Using nonverbal methods to assess paranoid ideation requires more investigation and evaluation. The integration of nonverbal assessments might offer new diagnostic possibilities that move beyond traditional verbal methods.

## Introduction

Paranoia is defined as a range of thoughts from mild worries that relate to social judgment to strong beliefs of reference and fear that an entity is intending to cause harm (Green et al., 2011). These beliefs range from paranoid ideations to persecutory delusions. Approximately one in three people in the general population experiences paranoid thinking to some extent during their lifetime (Freeman et al., 2011). Over 70% of people experiencing early stages of psychosis present with persecutory delusions (Coid et al., 2013). These delusions, held with strong conviction and resistant to contrary evidence significantly impact functioning, mood (Vorontsova, Garety, & Freeman, 2013), and sleep (Freeman, Pugh, Vorontsova, & Southgate, 2009). The severity of these beliefs, which is key to diagnosis, is determined by factors such as conviction, distress, impact on functioning, preoccupation, and resistance to change (Garety et al., 2005). Diagnosing paranoid ideation typically involves self-report or hetero-anamnestic evidence from interviews and questionnaires (such as the Positive and Negative Syndrome Scale (PANSS; Kay, Fiszbein, & Opler, 1987); Comprehensive Assessment of At-Risk Mental States (Yung et al., 2005); and Green Paranoid Thoughts Scale (Green et al., 2008). Interviews and questionnaires can potentially be suboptimal for diagnosing paranoia, as individuals may avoid sharing details due to mistrust or fear of judgment. Additional evidence for the diagnosis of persecutory delusions might be provided through clinical observation and other nonverbal measurements, although a synthesis of evidence for this in the literature is lacking. The aim of this article is to provide an overview of nonverbal correlates of paranoid ideation. Results can be used to further develop nonverbal components of diagnostic instruments in order to provide useful triangulation in clinical assessment.

Currently, people’s ability and willingness to verbally communicate are a vital part of diagnostic assessment. When this is hampered due to anxiety, suspiciousness, or other symptoms, the validity of the diagnostic process becomes vulnerable. Studies show that social interactions and relationships can be complicated for people experiencing paranoid delusions (Fett et al., 2022; Freeman et al., 2011; Gorisse et al., 2021). Furthermore, language and cultural contexts can add to the challenge of the diagnostic process and interpretation of symptoms. For example, behaviors that may appear paranoid, such as heightened mistrust or hypervigilance, can stem from real threats or from experiences with racism and oppression, rather than from clinical delusional ideations (Whaley, 2001). When the interaction between clinician and patient is disrupted, questions that assess symptom severity can become ineffectual. In these cases, healthcare professionals have to rely on records, context, and clinical experience to assess and formulate diagnoses. Research has shown some reliability in this approach of diagnosis (Ekholm et al., 2005). However, this diagnostic process is not standardized nor operationalized, which challenges its validity, especially in critical stages of the disorder.

Ideally, auxiliary methods in the form of observations of nonverbal correlates could provide supplemental evidence for diagnosis. Opportunities for assessing these nonverbal correlates exist on multiple facets. Anxiety and negative emotions impact the development and maintenance of persecutory delusions (Freeman & Garety, 2014). Negative emotional states have nonverbal correlates including posture (Oosterwijk, Rotteveel, Fischer, & Hess, 2009), facial expressions (Ekman, Freisen, & Ancoli, 1980), and breathing patterns (Masaoka & Homma, 1997). This type of additional information could potentially aid the diagnostic process compared to reliance on verbal reports alone. Additionally, cognitive biases associated with persecutory delusions, such as jumping-to-conclusions and bias against disconfirmatory evidence (Ho-wai So, Freeman, & Garety, 2008; Van Dael et al., 2006), could be revealed through nonverbal tasks. One way to do this is through assessment in decision-making tasks when minimal evidence is presented, when decisions are made faster, that is, with less information a tendency to jump-to-conclusions could be present (Catalan et al., 2022). Additionally, the perception of threat can trigger physiological and neurobiological reactions (Kozlowska, Walker, McLean, & Carrive, 2015). Finally, deficits in social cognition and emotion perception, which impair the ability to accurately interpret and respond to others’ mental states, emotions, and social cues, can be assessed nonverbally.

While studies exist that investigate nonverbal correlates, the literature is devoid of a systematic literature review of nonverbal correlates relating to paranoid ideation. To explore this topic, we conducted a systematic overview of the existing literature, with a specific focus on objectively measured correlates of a nonverbal nature. This review can help identify potential additional measures of paranoid ideations and methodological. Results may offer insights into the mechanisms of paranoid ideation and could inform the development of novel diagnostic instruments that do not depend solely on verbal communication, bridge language barriers, and potentially contributing to diagnostic accuracy.

## Methods

This systematic literature review was registered with PROSPERO (ID: CRD42022288001). The research protocol follows the Preferred Reporting Items for Systematic reviews and Meta-Analyses (PRISMA) guidelines (Page et al., 2021). The PRISMA checklist is added in Supplementary Material A. The PICO framework (participants, interventions, comparators, outcomes) was used to determine the eligibility criteria for this study, which resulted in the inclusion and exclusion criteria below. Databases were searched in May 2022 and a search update took place in April 2024.

### Inclusion and exclusion criteria

Studies were included if they met the following inclusion criteria: (1) use of standardized instruments for measurements of paranoid ideation; (2) use of standardized instrument for measurement of nonverbal correlate; (3) adult participants, aged 16 and older; (4) both clinical (delusions) and subclinical (paranoid ideations) levels of paranoia, (5) original data; and (6) published in a peer reviewed journal. Studies were excluded based on the following exclusion criteria: (1) nonverbal correlate is measured using a verbal method and (2) full text not available in English language.

No inclusion criteria were formulated around use of comparator group or study methodology, meaning articles with and without a control group were included as well as both qualitative and quantitative studies. No restrictions were placed on date of publication. No contextual restrictions for the studies were included in the review, allowing for a broad understanding of nonverbal correlates of paranoia in diverse settings.

### Search strategy

Four databases were used: PsycINFO, PubMed, Web of Science, and Cienahl. Our search strategy contained terms for paranoia (e.g. paranoid ideation, persecutory delusions) and nonverbal behavior (e.g. nonverbal communication, behavioral symptoms). The search strategy for each database can be found in Supplementary Material B. As a secondary search strategy, the titles in the reference lists of included papers were screened.

### Screening and data extraction

**Figure 1.**
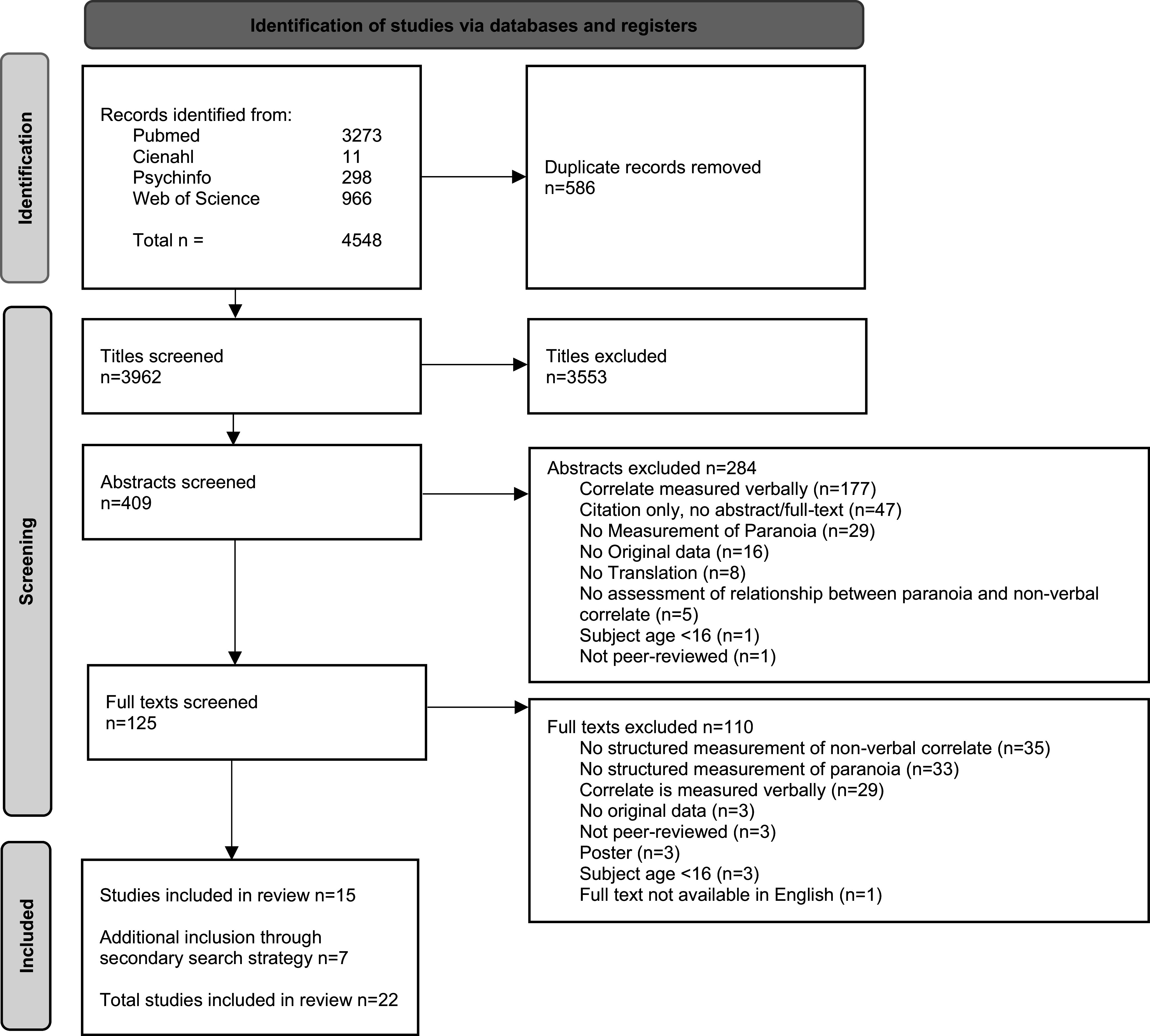
PRISMA flowchart.

Duplicates were removed and the remaining articles were independently screened by two of the authors (RH and CG/HJ) in three phases: title, abstract, and full text (see Figure 1). During each phase, assessment of eligibility criteria was conducted with any discrepancies resolved through discussion between all review authors. Data were systematically extracted from the selected studies using a data extraction sheet including: authors, year of publication, country of study, N, gender, diagnosis, used instruments, nonverbal concept being measured, and main outcomes. Main outcome data were extracted on any quantitative measure where possible, such as correlations, odd ratios, effect sizes, relative risks, and risk ratios. Similarly, qualitative data measures were extracted, for example: thematic and axiological analyses. Data were summarized thematically and narratively.

Meta-analyses were planned in case five or more studies with the same nonverbal correlate were found. This limit was chosen as literature has shown that estimating between-study variance with fewer than five studies can be unstable and unreliable (Jackson & Turner, 2017). Since there were no categories with minimally five studies, meta-analyses were not conducted

### Quality assessment

For the included studies, the ‘Effective Public Healthcare Panacea Project’ quality assessment (QA) tool was used (Thomas, Ciliska, Dobbins, & Micucci, 2004). The Effective Public Health Practice Project (EPHPP) was selected as it allows for a standardized assessment across diverse methodologies. Given the absence of restrictions on research design in the inclusion criteria, a variety of study designs was anticipated, making the EPHPP approach particularly suitable. QA was performed by two authors independently (RH and CG/HJ). QA criteria were: selection bias, study design, confounders, blinding, data collection methods, withdrawals and dropouts, intervention integrity, and analysis. Scores ranged from 1 (strong) to 3 (weak), thus higher value indicates a lower quality. Average quality scores were calculated, full sores can be found in Supplementary Material C.

### Paranoia concept formulation

Included studies show conceptual overlap concerning paranoid ideations but written terminologies have lexical variation between authors. In this review, the term paranoid ideation is used to offer uniform terminology.

## Results

The final search was executed in April 2024 and resulted in a total of 3962 titles after duplicate removal. After abstract and full-text screening, 15 studies met inclusion criteria. The secondary search strategy yielded 45 potential studies of which seven met inclusion criteria, resulting in a total of 22 studies included in this review (Figure 1). Main reasons for exclusion during full-text screening (*n* = 125) were a non-structured measurement of either paranoid ideation or the nonverbal correlate (*n* = 68, 54%), and verbal measurement of the nonverbal correlate (*n* = 29, 23%). Of the 22 included studies, 11 (50%) included patients, and the other 11 studies were conducted with a nonclinical sample. Combined the studies included a total of 1572 participants, of which 458 (29%) were patients diagnosed with a psychotic disorder.

Table 1 provides an overview of the included studies; these are grouped in categories of type of nonverbal concept. Due to heterogeneity of data, no formal data synthesis was possible. While interpretations of effect sizes (small, medium, large) are reported where available, it is important to note that many of the included studies did not provide standardized effect sizes. Identified nonverbal concepts were *spatial behavior* (*n* = 6)*, brain region activity* (*n* = 5)*, visual perception* (*n* = 5)*, stress physiology* (*n* = 4)*, information processing* (*n* = 3), and *aggression* (*n* = 1). Two included articles studied multiple nonverbal concepts and therefore reoccur in Table 1.

**Table 1.** Summary of Included Studies by Nonverbal Concept: Participants, Measures, Findings, and Quality Assessment

Study	Participants	Nonverbal–verbal measurement	Main findings	QA range 1–3
**Spatial behavior**				
Brown (2014), Turkey	• 28 Psychotic disorder (46% ♀)	Approach and avoidance behavior – PS	Greater avoidance of angry faces with an averted gaze was observed in patients with higher paranoid ideation, with a moderate effect size. No significant correlation was found for angry faces with a straight gaze.	2.7
Combs (2004), USA	• 29 Nonclinical, high paranoia group (76% ♀) • 31 Nonclinical, low paranoia group (61% ♀)	Interpersonal distance – PS	More distance (on average 31 cm) was kept from the examiner for the high paranoia group.	1.5
Diamond (2022), UK	• 75 Nonclinical sample (43% ♀)	Step count – GPTS	No difference was found between step activity profile and paranoid ideation	2.3
Fornells-Ambrojo (2016), UK	• 61 Nonclinical sample (0% ♀)	Interpersonal distance – PS	Larger interpersonal distance predicted higher levels of paranoid ideation.	1.8
Geraets (2018), Netherlands	• 50 Psychotic disorder (20% ♀) • 19 Ultrahigh Risk (63% ♀) • 40 Siblings (45% ♀) • 47 Control (53% ♀)	Interpersonal distance – SSPS	No relation between interpersonal distance and paranoid ideation within a virtual reality environment.	1.7
Schoretsanitis (2016), Switzerland	• 64 Psychotic disorder (47% ♀) o 13 Paranoid power (46% ♀) o 22 Neutral affect (27% ♀) o 29 Paranoid threat (62% ♀) • 24 Nonclinical sample (46% ♀)	Interpersonal distance – BPS (grouped in *paranoid threat* for negative scores, neutral scores, and *paranoid power* for positive scores) and PANSS (p6)	Interpersonal distance was more than twice as large for the paranoid threat patient group when compared to patients with paranoid power, neutral affect, and nonclinical. Increase personal distance was predicted by higher scores on the BPS affect rating and higher scores on the positive symptoms of the PANSS.	1.8
**Brain region activity**				
Bourne (2014), UK	• 124 Nonclinical sample (48% ♀)	Emotional processing – PSQ	Weaker right hemisphere lateralization with small to moderate effect sizes was shown for higher levels of paranoid ideation when processing fear and sad facial expressions, but only in males.	2.5
Decross (2020), USA	• 43 Nonclinical, high paranoia group (65% ♀) • 44 Nonclinical, low paranoia group (75% ♀)	Amygdala V1 connectivity – PDI (persecutory factor)	Excessive functional connectivity between the amygdala and visual cortex showed to be a possible indicator of paranoid ideation.	2.2
Fan (2021), USA	• 23 Paranoid patients (52% ♀) • 22 Non-paranoid patients (41% ♀)	Right amygdala functional connectivity – PS self-report	A large effect was found for the functional connectivity of the right amygdala, frontal and pre-frontal cortex, and right insula. This was positively correlated with paranoid ideation.	1.8
Pinkham (2022), USA	• 32 Nonclinical, paranoia induction (69% ♀) • 31 Nonclinical sample (74% ♀)	Increased resting activity of the amygdala – PS	No increased of cerebral blood flow (CBF) was shown in the paranoia induction group. Increased CBF overtime was found for the paranoia induction group for the right amygdala with a moderate effect size. This was in direct contrast to the decrease in amygdala CBF for the control group.	1.5
Walther (2022), Switzerland	• 49 Paranoid Schizophrenia (35% ♀) • 40 Non-paranoid Schizophrenia (40% ♀) • 76 Nonclinical sample (59% ♀)	Resting-State functional connectivity – PANSS (sum of P1, P4, P5, P6)	Heightened connectivity between the hippocampus and amygdala was found in patients with paranoid ideation, this correlated with symptom severity as well. Increased connectivity in prefrontal areas was found in patients with paranoid ideation.	2.2
**Visual perception**				
Hillmann (2015), Germany	• 28 Nonclinical, low paranoia group (64% ♀) • 21 Nonclinical, high paranoia group (76% ♀)	Scanpath – PC	When looking at neutral faces, fewer fixations for salient facial features were present in the high paranoid ideation group with a small effect size.	1.7
Hillmann (2017), Germany	• 22 Nonclinical, low paranoia group (75% ♀) • 28 Nonclinical, high paranoia group (64% ♀)	Scanpath – PC	Shorter scanpaths were present in the high paranoia group with small to moderate effect sizes. No differences were found for the amount of longer fixations and amount of fixations within the area of salient facial features (eyes, nose, and mouth).	1.7
Hillmann (2018), Germany	• 23 Psychotic disorder (41% ♀) • 22 Nonclinical sample (42% ♀)	Scanpath – PC	Reduced visual attention to salient facial features (eyes, nose, and mouth) of neutral faces correlated with higher levels of paranoid ideation.	2.0
Phillips (2000), UK	• 18 Psychotic disorder with paranoid delusions (16% ♀) • 8 Psychotic disorder without paranoid delusions (13% ♀) • 19 Nonclinical sample (33% ♀)	Scanpath – PANSS	Less time was spent focusing on potentially threatening foreground areas (i.e. areas containing the prominent activity and detail) of the ambiguous picture and more time on nonthreatening foreground areas in the paranoid delusions group. For neutral and overtly threatening scenes, no differences between groups were found.	1.8
Sanders (2012), Germany	• 31 Psychotic disorder with paranoia (29% ♀) • 31 Nonclinical sample (32% ♀)	Motion masking behavior – PANSS	Stronger motion masking (meaning the focus on an object is hampered because of movements around the object) was present in patients with moderate to large effect sizes. Both groups showed intact predictions of visual events.	1.8
**Stress physiology**				
Clamor (2018), Germany	• 59 Nonclinical sample (54% ♀)	HRV and HR – PC	Lower heart rate during a subsequent resting period was related to paranoid ideation after a social stressor was presented, with a moderate effect size. No relation to HRV was found.	2.0
Dobson (1989), UK	• 28 Episodic o 14 Paranoid (21% ♀) o 14 Non-paranoid (21% ♀) • 28 Remitted o 14 Paranoid (21% ♀) o 14 Non-paranoid (21% ♀) • 28 Nonclinical sample o 14 Paranoid (21% ♀) o 14 Non-paranoid (21% ♀)	HR – Maine scale for paranoid and non-paranoid schizophrenia	Faster heart rates were found in the episodic patient groups. There was no difference between non-paranoid ideation episodic patients and paranoid ideation episodic patients.	2.5
Schlier (2019), Germany	• 62 Nonclinical sample (72% ♀)	SCL and HRV – PC	Parasympathetic activity decreased before the start of a paranoid ideation episode. These patterns continued during the episodes but went away afterward. Afterward a delayed increase in sympathetic activity was measured by SCL.	2.2
Strakeljahn (2024), Germany	• 62 Nonclinical sample (74% ♀)	HR and HRV – PC	Heart rate and heart rate variability predicted paranoid ideation with an accuracy of 63%–70%. However, the model showed low positive predictive values: 29%–36%, indicating frequent false-positives.	2.2
**Information processing**				
Combs (2004), USA	• 29 Nonclinical, high paranoia group (76% ♀) • 31 Nonclinical, low paranoia group (61% ♀)	Time reading – PS	Twice as long time was spent to read a consent form in the high paranoia group.	1.5
Dobson (1989), UK	• 28 Episodic o 14 Paranoid (21% ♀) o 14 Non-paranoid (21% ♀) • 28 Remitted o 14 Paranoid (21% ♀) o 14 Non-paranoid (21% ♀) • 28 Nonclinical controls o 14 Paranoid (21% ♀) o 14 Non-paranoid (21% ♀)	Reaction time & active coping behavior (taps to reduce external manipulated stressor) – Maine scale for paranoid and non-paranoid schizophrenia	Faster tapping speed was observed in the patient group with paranoid ideation during the stressor trials when compared to the patient group without paranoid ideation, the patients with paranoid ideation did not differ from the non-patient control group.	2.5
Moritz (2007), Germany	• 24 Psychotic disorder (33% ♀) • 34 Nonclinical controls (47% ♀)	Reaction time – PANSS	Patients responded faster to targets following paranoia-relevant pictures compared to controls. No difference was found between patients with and without paranoid ideation.	2.0
**Aggression**				
van Dongen (2012), Netherlands	• 37 Schizophrenia (0% ♀) • 5 Schizo-affective disorder (0% ♀) • 2 Psychotic disorder NOS (0% ♀)	Aggression – PIQ	Observed aggressive behavior had a positive relationship with paranoid ideation with a moderate effect size. This relationship was mediated by delusional distress.	1.8

*Note:* BPS, Bern Psychopathology Scale; GPTS, Green Paranoid Thoughts Scale; HRV, Heart Rate Variability, HR, Heart rate; PANSS, Positive and Negative Syndrome Scale; PC, Paranoia checklist; PDI, Peters et al. Delusions Inventory; PIQ, Persecutory Ideation Questionnaire; PS, Paranoia Scale; PSQ, Paranoia/Suspiciousness Questionnaire; SCL, Tonic Skin Conductance Level; SSPS, State Social Paranoia Scale.

### Spatial behavior (*n* = 6)

Increased paranoid ideation seemed to be associated with increased physical interpersonal distance (Combs & Penn, 2004; Fornells-Ambrojo et al., 2016; Schoretsanitis et al., 2016) and avoidance of potentially threatening social cues (Brown et al., 2014) in both clinical and nonclinical samples. Combs and Penn (2004) found that the high paranoid ideation group maintained an average interpersonal distance that was 31 cm greater than those in the low paranoid ideation group. Schoretsanitis et al. (2016) found interpersonal distance increased more than twofold for patients in the paranoid threat group compared to patients in the paranoid power group (i.e. delusion of grandiosity) and controls. Brown et al. (2014) showed a moderate positive relationship between paranoid ideation and avoidance of potentially threatening social cues. Both Fornells-Ambrojo et al. (2016) and Geraets et al. (2018) looked at personal distance in a virtual environment. Results were mixed, Fornells-Ambrojo et al. (2016) found persecutory ideation predicted increased interpersonal distance, Geraets et al. (2018) found no relationship between persecutory ideation and interpersonal distance. Finally, a study looking at activity profiles of patients (consisting of step count, meaningful activity, and mobility issues) showed no relationship with level of persecutory ideation and activity profile group (Diamond et al., 2022).

### Brain region activity (*n* = 5)

The studies on neurobiological correlates of paranoid ideation showed that the amygdala is significantly involved as a key brain area in threat processing. Amygdala activity was amplified (Fan et al., 2021) and an increased connectivity was found to the visual cortex (Decross et al., 2020) and to the hippocampus (Walther et al., 2022) in individuals with high levels of paranoid ideation. Both when comparing high and low levels of paranoid ideation in healthy samples (Decross et al., 2020) and when comparing controls with patients with paranoid ideation and schizophrenia, with Fan et al. (2021) reporting a large effect size (Fan et al., 2021; Walther et al., 2022). In a nonclinical sample, Bourne and McKay (2014) showed weaker right hemisphere lateralization in males when processing fear and sad facial expressions when paranoid ideation was higher. Pinkham et al. (2022) showed an increase in connectivity in the prefrontal cortex for individuals with higher level of paranoid ideation.

### Visual perception (*n* = 5)

Individuals with higher levels of paranoid ideation might exhibit distinct visual perception patterns. Included papers mainly looked at scanpath behavior and visual fixations. A visual scanpath refers to the amount of saccades (small eye movements) a person makes when looking at different parts of a scene or image. Meaning a reduced scanpath shows fewer eye movements (Hillmann, Ascone, Kempkensteffen, & Lincoln, 2017). Visual fixation occurs when the eyes stay focused on a specific point for more than 100 m/s (Hillmann, Kempkensteffen, & Lincoln, 2015). Differences for visual perception were apparent in both clinical and nonclinical samples. Paranoid ideation seemed to be related to reduced scanpaths, with a small to medium effect size reported (Hillmann, Ascone, Kempkensteffen, & Lincoln, 2017), reduced visual attention to salient facial features of the eyes, mouth, and nose, with a small effect size reported (Hillmann, Kempkensteffen, & Lincoln, 2015; Hillmann, Riehle, & Lincoln, 2018), and reduced visual attention of photograph areas containing activity and detail in ambiguous social scenes (Phillips, Senior, & David, 2000). Higher paranoid ideation can also affect visual processing when looking at dynamic environments. When a visual cue is moving or when it is presented in a visually complex environment, it becomes complicated to separate important details from surrounding distractions, this is called motion masking. This effect was shown to be stronger for patients with persecutory ideations, with a medium to large effect size reported (Sanders et al., 2012). Within nonclinical samples mixed results were found for the association of paranoid ideation and visual fixations Hillmann, Kempkensteffen, & Lincoln, 2015; Hillmann, Ascone, Kempkensteffen, & Lincoln, 2017). However, the study by Phillips, Senior, and David (2000) with clinical sample patients with paranoid ideation showed fewer fixations and different focus of fixations compared with controls.

### Stress physiology (*n* = 4)

No evidence of an association in clinical samples was found between stress physiology and paranoid ideation (Dobson & Neufeld, 1989). Looking at levels of paranoid ideation in nonclinical samples, the results showed low predictive value of physiological indicators, such as heart rate variability (HRV) and heart rate (HR) (Clamor & Krkovic, 2018; Strakeljahn, Lincoln, Krkovic, & Schlier, 2024). Schlier, Krkovic, Clamor, and Lincoln (2019) showed patients had a lower HR and a decrease in parasympathetic activity before a paranoid episode.

### Information processing (*n* = 3)

Patients were shown on average to spend more than twice as long reading an informed consent form (65 s compared to 128 s) for participation in research, which seems to indicate slower and possible more cautious information processing in neutral or nonurgent situations in individuals with higher levels of paranoid ideation (Combs & Penn, 2004). A study by Dobson and Neufeld (1989) did not find differences in responses on a stressor task: subjects were instructed to control the length of a noise played through headphones by tapping the index finger of their preferred hand quickly. Moritz and Laudan (2007) did find patients responded faster to paranoia relevant pictures, but did not find differences between patients with and without paranoid ideation.

### Aggression (*n* = 1)

The study by van Dongen, Buck, and van Marle (2012) showed that paranoid ideation had a moderate positive relationship with observed aggressive behavior, which was mediated by delusional distress.

### Quality assessment

Overall, the studies demonstrated strong performance in addressing confounders and employing robust data collection methods, while showing weaker scores in selection bias and blinding. Eleven of the included articles were of moderate to good quality (*n* = 50%), three of moderate quality (n=14%), and eight of moderate to weak quality (*n* = 36%) (see Table 1).

## Discussion

This is the first systematic literature review on nonverbal correlates of paranoid ideation. This review included 22 studies that examined 5 categories of nonverbal correlates associated with paranoid ideation. First, the results for each identified category will be discussed.

### Spatial behavior (*n* = 6)

In essence, results suggest that paranoid ideation influences physical distancing and threat avoidance, highlighting how it alters social behavior. According to the cognitive model of Freeman (2016), persecutory delusions are conceptualized as threat beliefs, which appear to drive increased personal distance as a protective mechanism. In this model, safety behaviors maintain paranoid ideation as it prevents exposure to contradictory evidence, which make them a possible target for interventions (Berkhof et al., 2024). However, spatial safety behavior might be modulated by different contextual variables such as environmental familiarity or how busy an environment is. Subsequently, results from studies on spatial behavior in virtual reality show that personal distance is possibly regulated differently in a virtual environment. Not all dimensions of spatial behavior seem to be related to paranoid ideation, as seen by a lack of support for a relationship between paranoid ideation and activity profiles (Diamond et al., 2022). Results support a threat-avoidance model, where higher paranoid ideation leads to modification of the physical environment to feel safer. However, larger, controlled studies are needed. It remains unclear to what extent spatial behavior is being modulated by factors, such as the social environment, familiarity, or specific stressors.

### Brain region activity (*n* = 5)

Results underpin paranoid ideation’s integration of emotional and cognitive responses. Increased amygdala activation paralleled with heightened prefrontal connectivity suggests the existence of two neural pathways within paranoia: the amygdala is involved in fast threat detection while the prefrontal cortex contributes to rational decision-making and the control of thought patterns related to paranoid ideation. This seems to involve cognitive effort in managing emotional responses. The specific role of the amygdala and prefrontal cortex in paranoia appears to fit within the general framework of psychosis-related neural activity (Feola et al., 2024) wherein the amygdala has shown to be pivotal as part of the neural mechanism in fear and anxiety, with specific associations for psychosis with the hippocampus and ventromedial prefrontal cortex.

### Visual perception (*n* = 5)

Visual perception and specifically gazing behavior has been studied extensively in patients with a psychotic disorder. Summarizing these results in a recent scoping review Wolf, Ueda, and Hirano (2020) concluded that longer average eye fixation, fewer total eye fixations, and shorter average scanning length are present in patients with schizophrenia. These results overlap with studies looking at paranoid ideation and gazing behavior. In terms of theoretical underpinning, the vigilance-avoidance paradigm of paranoia (Green & Phillips, 2004) offers an explanation for the deviation gazing behavior, as mentioned by Hillmann, Riehle, and Lincoln (2018).Individuals with higher paranoid ideation initially show increased bias toward potentially threatening information, which is followed by avoidant behavior in the form of reduced visual fixation on relevant areas. This avoidant behavior is shown to be present for individuals with higher paranoid ideation when presented with neutral faces. However, to which extent this explanation accounts specifically for paranoid ideation or is more general applicable to the broader spectrum of schizophrenia remains unclear. Social context seemingly plays a role in visual scanning behavior, since ambiguity of visual stimuli (either facial or environmental) influences how patients and nonclinical individuals with higher levels of paranoid ideation pay visual attention. Summarized, results suggest that paranoid ideation leads to changes in visual perception, which influences scanning behavior, visual fixation, and motion masking, these behaviors fluctuate with clinical status and social context.

### Stress physiology (*n* = 4)

The lack of consistent physiological markers poses a challenge to simple biological models of paranoid ideation. Physiological measures such as HR and HRV, commonly used to assess autonomic nervous system activation, represent a general measure of arousal influenced by a wide range of factors – both negative, positive, and neutral. This complexity makes it difficult to isolate the direct impact of paranoid ideation. If such markers are to be useful, their application would likely require integration with other complementary measures to provide a more comprehensive understanding. Reactions could be seen as part of the body’s context-dependent response to perceived threats (Kozlowska, Walker, McLean, & Carrive, 2015). Which would underscore the theory’s emphasis on the cascading nature of threat responses, influenced by multiple factors beyond just paranoia. As proposed by Schlier, Krkovic, Clamor, and Lincoln (2019), one hypothesis is that paranoid thinking works as a coping mechanism to deal with stress due to perceived threat. In that case, hypervigilance might cause physiological threat reactions (i.e. activation of sympathetic nervous system) but the existence of paranoid thoughts might cause parasympathetic reactions. This would explain different findings among the presented studies. Since responses vary widely, relying on physiological markers for diagnostic purposes may be limited by a lack of sufficient information at this point.

### Information processing (*n* = 3)

Individuals diagnosed with a psychotic disorder may exhibit selective sensitivity to stimuli that align with paranoid thoughts but not a generalized change in processing speed. This might reflect the hyper vigilant state of paranoia, where one shows heightened suspicion when dealing with neutral stimuli. Heightened suspicion or threat perception influences cognitive and emotional processing, even for seemingly neutral stimuli. This aligns with previous explanation that paranoid ideation integrates emotional and cognitive responses through two distinct but interrelated neural pathways. These reflect the cognitive effort required to manage emotional responses, a dynamic central to paranoid ideation. However, as Feola et al. (2024) note, the roles of the amygdala and prefrontal cortex likely fall within the broader framework of psychosis-related neural activity, suggesting these mechanisms may not be exclusive to paranoid ideation but could extend to schizophrenia more generally.

### Aggression (*n* = 1)

Although a moderate positive relationship was found for aggressive behavior and paranoid ideation, results come from one study. The particular study focused on a male sample of forensic clinical patients. Based on the specificity of this setting and low *n*, generalization of findings is not possible at this point in time.

Results of this systematic literature review reveal that investigations on nonverbal correlates of paranoid ideation are scarce, quite heterogeneous in approach and populations, and limited by small sample sizes. Nevertheless, measures in several domains showed statistical, clinically relevant, and theoretically plausible associations with paranoid ideations. The potential for nonverbal correlates to be used diagnostically is an interesting field, although in order to enhance diagnostic accuracy, probably the combination of both nonverbal and verbal signs is needed. Future research should focus on developing frameworks that integrate both types of indicators. Theoretically, this integration could provide a more holistic understanding of paranoid ideation, capturing both explicit verbal expressions and implicit nonverbal signals. However, given the current limitations in research, further exploration is needed to better understand how these two forms of assessment might complement each other in practice.

When comparing clinical and nonclinical populations, findings on spatial behavior and brain region activity suggest similar patterns, which could mean underlying mechanisms of paranoid ideations are shared. In terms of visual attention, results suggest that paranoid ideation is associated with changes in visual perception both in clinical and nonclinical populations. Stress physiology related to paranoid ideations has hardly been studied in clinical populations, with only one out of four studies including patients, making it difficult to determine whether stress responses differ between clinical and nonclinical groups. In terms of information processing, nonclinical subjects with high paranoid ideation took longer to read consent forms, whereas clinical subjects with high levels of paranoid ideation showed quicker responses in task-related measures compared to those with lower level of paranoid ideations. Due to the limited evidence, it remains unclear to what extent clinical and nonclinical groups differ in the nonverbal correlates of paranoia. However, the available findings suggest some overlapping patterns that require further investigation.

Finally, in some populations, nonverbal measures may be particularly useful, for example, when people cannot or will not speak, in patients with intellectual disabilities or with other communication barriers. Furthermore, investigating how contextual variables – such as social settings, environmental, or stress-inducing conditions – modulate these nonverbal correlates need to be assessed in order to ascertain the situational dynamics of paranoid ideation. Controlled virtual reality experiments may be particularly suited to test such associations. This can potentially identify predictive factors or early markers for paranoia, which can contribute to improving clinical outcomes through earlier and more accurate detection and targeted treatment strategies.

### Strengths and limitations

A key strength of first systematic review into nonverbal correlates of paranoia lies in its strict inclusion criteria, excluding studies relying on verbal components. By doing so, the review reduced potential confounding factors associated with verbal elements. Subsequently, this approach narrowed the pool of eligible studies, providing a more precise exploration of the specific nonverbal correlates addressed. This helped increase precision of the synthesized evidence to stay close to the relevant review’s objectives. Nevertheless, findings should be interpreted in light of the following limitations: First, a majority of the included studies rely on small sample sizes and only half of the included papers investigated participants with clinical levels of paranoia. Therefore, results do not capture the full spectrum of paranoid ideation and subgroup variations in terms of severity or comorbidity, negatively impacting on scope and generalizability. The small sample sizes reduce reliability of findings and generalizability. It is also worth noting that the majority of included studies reported significant associations between paranoid ideation and nonverbal correlates. Although we did not conduct a formal analysis of publication bias, this uneven distribution may suggest a potential bias in favor of publishing significant findings. Second, the categories of nonverbal concepts studied are heterogeneous, as are the definitions of paranoid ideation. Within the presented categories a broad range of experimental paradigms was found. For example, experiments investigating visual fixation patterns all had slight variations in their methodological approach. This complicates synthesis of results and impacts the strength of given conclusions and its robustness. Subsequently, although a meta-analysis was planned, it was not conducted due to an insufficient number of studies per category of nonverbal correlates. Third, self-report on nonverbal correlates or informant report on nonverbal correlates were not included in the review due to reliance on a verbal instrument. Studies show that safety behavior plays an important role in paranoid ideation (Freeman, Garety, & Kuipers, 2001); however, there were no studies available that gathered data nonverbally. It is possible that similar other seemingly nonverbal correlates are excluded due to the usage of verbal measurements. Fourth, it cannot be ruled out that the nonverbal correlates identified by this review might reflect broader symptoms of psychosis or threat. The possibility that these correlates lack specificity toward paranoid ideation reduces the precision of findings. On the other hand, certain nonverbal correlates of threat responses might also coalesce with paranoia.

## Conclusion

Spatial behavior and brain region activity were shown to have the most potential as possible nonverbal indicators of paranoid ideation, whereas findings on visual processing remain inconclusive due to methodological differences. Evidence for stress physiology, information processing, and aggression as potential nonverbal correlates was less robust. Further investigation of the identified categories is needed, potentially leading to new diagnostic and therapeutic possibilities that move beyond traditional verbal methods.

## Supporting information

Haarms et al. supplementary material Haarms et al. supplementary material

## Supplementary material

The supplementary material for this article can be found at http://doi.org/10.1017/S0033291725001230.
