# Supplementary material for: Nonverbal correlates of paranoid ideation – a systematic literature review
Source: Psychol Med. 2025 Jun 4;55:e165. doi: 10.1017/S0033291725001230 (PMC12180505; doi:10.1017/S0033291725001230)
Supplement: Haarms et al. supplementary material [file S0033291725001230sup001.zip › Supplement B-Search Strategy.docx]

**Supplement B**

Search strategy

Date of search strategy 22-4-2024

**PUBMED**

("Paranoid Disorders"[Mesh] OR "Paranoid Behavior"[Mesh] OR "paranoid disorder*"[tiab] OR "paranoid psychos*"[tiab] OR paranoia*[tiab] OR "paranoid behavior*"[tiab] OR "paranoid behaviour*"[tiab] OR "paranoid ideation*"[tiab] OR "paranoid personality disorder*"[tiab] OR "persecutory delusions*"[tiab])

(n=7,082)

AND

("fixation, ocular"[Mesh] OR "kinesics"[Mesh] OR "Posture"[Mesh] OR "Behavioral symptoms"[Mesh] OR "Nonverbal communication"[Mesh] OR "Dangerous behavior"[Mesh] OR "Escape reaction"[Mesh] OR "Information seeking behavior"[Mesh] OR "Exploratory behavior"[Mesh] OR "Motor activity"[Mesh] OR " Visual perception"[Mesh] OR "Spatial behavior"[Mesh] OR "Psychophysiology"[Mesh] OR "eyecontact"[tiab] OR "EyeMovements"[tiab] OR "ocular focusing"[tiab] OR "ocular fixation"[tiab] OR "eye gaze*"[tiab] OR "kinesics"[tiab] or "gesture*"[tiab] OR "bodylanguage"[tiab] OR "safety behav*"[tiab] OR "safety seeking behav*"[tiab] OR "visual scan*"[tiab] OR "visual processing"[tiab] OR "personal space regulation"[tiab] OR "approach-avoidance behavio*"[tiab] OR "fixation, ocular*"[tiab] OR "kinesics*"[tiab] OR "postur*"[tiab] OR " Behavioral symptom*"[tiab] OR " Behavioural symptom*"[tiab] OR "nonverbal communication"[tiab] OR "Dangerous behavio*"[tiab] OR "Escape reaction"[tiab] OR "Exploratory behavio*"[tiab] OR "Information seeking behavio*"[tiab] OR "Motor activity"[tiab] OR" Visual perception"[tiab] OR "Social behavior"[Mesh] OR "Social behavio*"[tiab] OR "Spatial behaviour"[tiab] OR "Psychophysiology"[tiab] OR "Arousal"[tiab] OR "biofeedback"[tiab] OR "blushing"[tiab] OR "orientation"[tiab] OR "reaction time"[tiab] OR "reflex"[tiab] OR "sensation"[tiab] OR "stress"[tiab] OR "mind-body relation*"[tiab])

(n=2,941,872)

Result combined: n=3,143

**CIENAHL**

((MH "Paranoid Disorders+") OR TI ("paranoid disorder*" OR "paranoid psychos*" OR paranoia* OR "paranoid behavior*" OR "paranoid behaviour*" OR "paranoid ideation*" OR "paranoid personality disorder*" OR "persecutory delusions*") OR AB ("paranoid disorder*" OR "paranoid psychos*" OR paranoia* OR "paranoid behavior*" OR "paranoid behaviour*" OR "paranoid ideation*" OR "paranoid personality disorder*" OR "persecutory delusions*"))

(n=1,603)

AND

((MH "Visual Perception+") OR (MH "Spatial behaviour+") OR (MH "Social behaviour+") OR (MH "Motor activity+") OR (MH "Information seeking behaviour+") OR (MH "Posture+") OR (MH body language+") OR (MH "Behavioral symptoms+") OR (MH "Nonverbal communication+") OR (MH "Risk Taking behaviour+") OR (MH "Psychophysiology+") OR TI ("eyecontact" OR "EyeMovements" OR "ocular focusing" OR "ocular fixation" OR "eye gaze*" OR "kinesics" OR "gesture*" OR "bodylanguage" OR "safety behav*" OR "safety seeking behav*" OR "visual scan*" OR "visual processing" OR "personal space regulation" OR "approach-avoidance behavio*" OR "fixation, ocular*" OR "kinesics*" OR "postur*" OR "Behavioral symptom*" OR " Behavioural symptom*" OR "nonverbal communication" OR "Dangerous behavio*" OR "Escape reaction" OR "Exploratory behavio*" OR "Information seeking behavio*" OR "Motor activity" OR "Visual perception" OR "Social behavio*" OR "Spatial behaviour" OR "Risk Taking behavio*" OR "Psychophysiology") OR AB ("eyecontact" OR "EyeMovements" OR "ocular focusing" OR "ocular fixation" OR "eye gaze*" OR "kinesics" OR "gesture*" OR "bodylanguage" OR "safety behav*" OR "safety seeking behav*" OR "visual scan*" OR "visual processing" OR "personal space regulation" OR "approach-avoidance behavio*" OR "fixation, ocular*" OR "kinesics*" OR "postur*" OR "Risk Taking behavio*" OR " Behavioral symptom*" OR "Behavioural symptom*" OR "nonverbal communication" OR "Dangerous behavio*" OR "Escape reaction" OR "Exploratory behavio*" OR "Information seeking behavio*" OR "Motor activity" OR" Visual perception" OR "Social behavio*" OR "Spatial behaviour" OR "Psychophysiology" OR "Arousal" OR "biofeedback" OR "blushing" OR "orientation" OR "reaction time" OR "reflex" OR "sensation" OR "stress" OR "mind-body relation*"))

(n=63,848)

Result combined: n=10

**PSYCHINFO**

(1)

DE "Paranoia" OR DE "Paranoia (Psychosis)" OR DE "Paranoid Personality Disorder" OR DE "Paranoid Schizophrenia"

(2) (keyterms)

"Paranoia" OR "Paranoia (Psychosis)" OR "Paranoid Personality Disorder" OR "Paranoid Schizophrenia" OR "paranoia*" OR "paranoid disorder*" OR "paranoid psychos*" OR paranoia* OR "paranoid behavior*" OR "paranoid behaviour*" OR "paranoid ideation*" OR "paranoid personality disorder*" OR "persecutory delusions*"

(3)

DE "Eye contact" OR DE "Eye Fixation" OR DE "Eye movements" OR DE "Body language" OR DE "visual search" OR DE "personal space" OR DE "approach avoidance" OR DE "posture" OR DE "nonverbal communication" OR DE "information seeking" OR DE "hyperkinesis" OR DE "visual perception" OR DE "social behavior" OR DE "spatial navigation" OR DE "psychophysiology"

(4) (keyterms)

"eyecontact" OR "EyeMovements" OR "ocular focusing" OR "ocular fixation" OR "eye gaze*" OR "kinesics" or "gesture*" OR "bodylanguage" OR "safety behav*" OR "safety seeking behav*" OR "visual scan*" OR "visual processing" OR "personal space regulation" OR "approach-avoidance behavio*" OR "fixation, ocular*" OR "kinesics*" OR "postur*" OR " Behavioral symptom*" OR " Behavioural symptom*" OR "nonverbal communication" OR "Dangerous behavio*" OR "Escape reaction" OR "Exploratory behavio*" OR "Information seeking behavio*" OR "Motor activity" OR" Visual perception" OR "Social behavio*" OR "Spatial behaviour" OR "Psychophysiology" OR "Arousal" OR "biofeedback" OR "blushing" OR "orientation" OR "reaction time" OR "reflex" OR "sensation" OR "stress" OR "mind-body relation*"

S 1 (n= 4.927)

S 2 (n=3.437)

S 3 (n=181,434)

S 4 (n=228,234)

Search strategy (S1 or S2) and (S3 or S4) n= 253

**WEB OF SCIENCE**

("Paranoia" OR "Paranoia (Psychosis)" OR "Paranoid Personality Disorder" OR "Paranoid Schizophrenia" OR "paranoia*" OR "paranoid disorder*" OR "paranoid psychos*" OR paranoia* OR "paranoid behavior*" OR "paranoid behaviour*" OR "paranoid ideation*" OR "paranoid personality disorder*" OR "persecutory delusions*")

(n=6,890)

And

("eyecontact" OR "EyeMovements" OR "ocular focusing" OR "ocular fixation" OR "eye gaze*" OR "kinesics" or "gesture*" OR "bodylanguage" OR "safety behav*" OR "safety seeking behav*" OR "visual scan*" OR "visual processing" OR "personal space regulation" OR "approach-avoidance behavio*" OR "fixation, ocular*" OR "kinesics*" OR "postur*" OR " Behavioral symptom*" OR " Behavioural symptom*" OR "nonverbal communication" OR "Dangerous behavio*" OR "Escape reaction" OR "Exploratory behavio*" OR "Information seeking behavio*" OR "Motor activity" OR" Visual perception" OR "Social behavio*" OR "Spatial behaviour" OR "Psychophysiology" OR "Arousal" OR "biofeedback" OR "blushing" OR "orientation" OR "reaction time" OR "reflex" OR "sensation" OR "stress" OR "mind-body relation*")

(n=2,875,467)

Result combined: n=880
