# Supplementary material for: Nonverbal correlates of paranoid ideation – a systematic literature review
Source: Psychol Med. 2025 Jun 4;55:e165. doi: 10.1017/S0033291725001230 (PMC12180505; doi:10.1017/S0033291725001230)
Supplement: Haarms et al. supplementary material [file S0033291725001230sup001.zip › Supplement C-Quality Assessment.docx]

**Supplement C**

**EPHPP Quality scores**

|  | Selection bias | Study design | Confounders | Blinding | Data collection method | Withdrawals and dropout | Global rating |
| --- | --- | --- | --- | --- | --- | --- | --- |
| Bourne et al. 2014 | 3 | 2 | 2 | 2 | 3 | 3 | 2,5 |
| Brown et al. 2014 | 3 | 3 | 2 | 3 | 2 | 3 | 2,7 |
| Clamor et al. 2018 | 3 | 2 | 3 | 1 | 2 | 1 | 2,0 |
| Combs et al. 2004 | 3 | 2 | 1 | 1 | 1 | 1 | 1,5 |
| Decross et al. 2019 | 3 | 3 | 1 | 3 | 2 | 1 | 2,2 |
| Diamond et al. 2022 | 2 | 3 | 3 | 3 | 2 | 1 | 2,3 |
| Dobson et al. 1989 | 3 | 2 | 3 | 3 | 1 | 3 | 2,5 |
| Fan et al. 2021 | 2 | 2 | 1 | 3 | 2 | 1 | 1,8 |
| Geraets et al. 2018 | 3 | 2 | 2 | 1 | 1 | 1 | 1,7 |
| Hillmann et al. 2015 | 2 | 2 | 1 | 3 | 1 | 1 | 1,7 |
| Hillmann et al. 2017 | 3 | 2 | 1 | 2 | 1 | 1 | 1,7 |
| Hillmann et al. 2018 | 2 | 2 | 2 | 3 | 1 | 2 | 2,0 |
| Moritz et al. 2007 | 3 | 1 | 1 | 3 | 3 | 1 | 2,0 |
| Phillips et al. 2000 | 3 | 2 | 1 | 3 | 1 | 1 | 1,8 |
| Pinkham et al. 2022 | 2 | 1 | 1 | 2 | 2 | 1 | 1,5 |
| Sanders et al. 2012 | 3 | 2 | 1 | 3 | 1 | 1 | 1,8 |
| Schlier et al. 2019 | 3 | 2 | 3 | 3 | 1 | 1 | 2,2 |
| Schoretsanitis et al. 2016 | 3 | 2 | 1 | 3 | 1 | 1 | 1,8 |
| Strakeljahn et al. 2024 | 3 | 3 | 3 | 2 | 1 | 1 | 2,2 |
| Fornells-Ambrojo et al. 2016 | 2 | 2 | 2 | 2 | 1 | 2 | 1,8 |
| van Dongen et al. 2012 | 2 | 2 | 2 | 3 | 1 | 1 | 1,8 |
| Walther et al. 2022 | 2 | 2 | 1 | 2 | 3 | 3 | 2,2 |
